# Epithelial dynamics shed light on the mechanisms underlying ear canal defects

**DOI:** 10.1242/dev.194654

**Published:** 2020-12-14

**Authors:** Juan M. Fons, Mona Mozaffari, Dean Malik, Abigail R. Marshall, Steve Connor, Nicholas D. E. Greene, Abigail S. Tucker

**Affiliations:** 1 Centre for Craniofacial and Regenerative Biology, King's College London, London SE1 9RT, UK; 2 Great Ormond Street Institute of Child Health, University College London, London WC1N 1EH, UK; 3 King's College Hospital NHS Foundation Trust, London SE5 9RS, UK; 4 School of Biomedical Engineering and Imaging Sciences Clinical Academic Group, King's College London, London SE1 9RT, UK

**Keywords:** Hearing loss, External ear, Canal atresia, Periderm

## Abstract

Defects in ear canal development can cause severe hearing loss as sound waves fail to reach the middle ear. Here, we reveal new mechanisms that control human canal development and highlight for the first time the complex system of canal closure and reopening. These processes can be perturbed in mutant mice and in explant culture, mimicking the defects associated with canal atresia. The more superficial part of the canal forms from an open primary canal that closes and then reopens. In contrast, the deeper part of the canal forms from an extending solid meatal plate that opens later. Closure and fusion of the primary canal was linked to loss of periderm, with failure in periderm formation in *Grhl3* mutant mice associated with premature closure of the canal. Conversely, inhibition of cell death in the periderm resulted in an arrest of closure. Once closed, re-opening of the canal occurred in a wave, triggered by terminal differentiation of the epithelium. Understanding these complex processes involved in canal development sheds light on the underlying causes of canal atresia.

## Abstract (precis)

**Highlighted Article:** We reveal new mechanisms that control development of the ear canal and highlight for the first time the complex system of canal closure and reopening.

## INTRODUCTION

The external ear canal funnels sound towards the ear-drum and middle ear. Defects in initiation, extension or opening of the canal cause canal aplasia (absence) or atresia/stenosis (malformation/narrowing), leading to deafness and hearing loss (Schuknecht, 1975). The external ear canal is supported internally by bone and cartilage. The bony part is associated with the auditory bulla that encloses the middle ear, while the cartilaginous part is more superficial leading out towards the pinna. In cases of aplasia/atresia (one in 10,000-15,000 births) canal reconstruction is sometimes attempted (atresiaplasty); however, such surgery has a fairly poor history of success, hampered by the fact that the surgically constructed epithelium often collapses, fuses and becomes infected (Edfeld and Stromback, 2015). Such operative management of congenital aural atresia is one of the most challenging surgeries faced by otologists as the operation is hindered by the unpredictability and variability of deformities of the canal (Abdel-Aziz, 2013). Currently, in the absence of a clear understanding of ear canal development, an observational CT-based grading system, focusing on the extent of associated ear deformities, is used to predict the success of canaloplasty surgery and choose candidate patients for surgery (Jahrsdoerfer et al., 1992). Although canal atresia and aplasia are linked to deformities of the pinna, middle and, less frequently, inner ear, their extent and severity are typically unpredictable. As a result, even with modern imaging, the operating surgeon is often presented with unexpected anatomy, increasing the chance of complications. The lack of an ear canal is therefore often simply bypassed by a bone-anchored hearing aid (BAHA) (Nadaraja et al., 2013). Implantable hearing devices have become a realistic alternative to BAHA in recent years but the complex and variable anatomy of the external and middle ear in individuals with aural atresia prevents their widespread use (Lo et al., 2014). In order to improve upon current devices and surgical techniques, a better understanding of the biological processes regulating development of the canal epithelium are needed. In this article we have investigated how the ear canal forms in human and mouse embryos, using mutant mice and explant culture to understand the mechanisms behind canal defects.

The simple final structure of the canal belies a complex system of development. The canal starts to form as an invaginating epithelial groove at around E (embryonic day) 12.0 in mouse and 44 days in human, and has a lumen (Michaels and Soucek, 1989, 1991). The leading tip of this open groove consists of a solid plug of epithelial tissue, known as the meatal plug, and as such the ear canal is often referred to as the external auditory (or acoustic) meatus (EAM). The meatal plug elongates towards the developing ear drum as a thin solid sheet of epithelium and creates the deeper part of the ear canal and the ectodermal lining of the three layered ear drum (Michaels and Soucek, 1991; Nishimura and Kumoi, 1992). The meatus then opens, postnatally in the mouse, to create the ear canal (Nishizaki et al., 1998). In humans the ear canal has been described as opening from 21-28 weeks, but has more recently been shown to be patent at 16 weeks (Nishimura and Kumoi, 1992). Human ear canal development has been described morphologically (Michaels and Soucek, 1989; Nishimura and Kumoi, 1992; Declau, 1995) but a clear understanding of the process has been hampered by reduced availability of tissue and a complete lack of molecular analysis. Here, we highlight a dynamic system of closure, extension and opening of the canal not previously appreciated, and use mouse models to test the mechanisms underlying ear canal defects.

## RESULTS

### The ear canal is formed from a primary canal and extension of a meatal plug

**Fig. 1.**
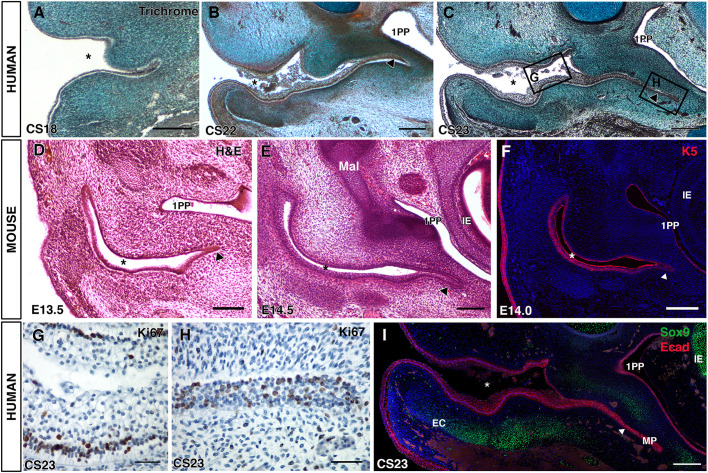
**The ear canal forms in two distinct parts.** (A-C) Trichrome-stained frontal sections through the developing human ear canal. (A) CS18, the meatus is just visible at the end of the open primary canal (asterisk). (B) CS22, the meatus (arrowhead) has extended towards the middle ear and first pharyngeal pouch (1PP). (C) CS23, the meatus has extended adjacent to the pharyngeal pouch as a thin sheet (arrowhead). (D-F) Forming and extending meatal plug, mouse frontal sections stained with Eosin (D,E) and for keratin 5 (F). (D) At E13.5 the meatal plug (arrowhead) is only evident as a small group of cells at the end of the open primary canal. (E) At E14.5, the meatal plug has extended towards the middle ear (arrowhead). (F) Keratin 5 expression at E14.0 in the primary canal (asterisk) and meatus (arrowhead). (G,H) Ki67 in human frontal sections. Images show regions indicated by rectangles in C. (G) The open primary canal with positive (brown) cells located at the basal layer. (H) The meatal plug with positive (brown) cells throughout the epithelium. (I) CS23, immunofluorescence for Sox9 (green) and E-cadherin (pink). DAPI nuclear stain (blue). The two parts of the canal [primary canal and meatal plate (MP)] are clearly visible. The developing external ear canal cartilages (ECs) are observed forming under the primary canal but not the meatal plate. Asterisks in A-F,I indicate the open primary canal. Scale bars: 500 µm in A-C; 200 µm in D-F,I; 100 µm in G,H. 1PP, first pharyngeal pouch; IE, inner ear; EC, ear canal cartilage; MP, meatal plate.

In human embryos, the external auditory canal has been described as initially forming from a primary canal at 44 days of development, Carnegie stage (CS) 18 (Michaels and Soucek, 1989). By CS18, the primary ear canal was evident as an open invagination of the surface ectoderm (Fig. 1A). In mouse and humans the tip of the primary canal, which extends into the first arch, forms a plug of epithelial cells that elongates towards the middle ear (Michaels and Soucek, 1989, 1991; Mallo and Gridley, 1996). To follow the timing of this event, we studied the development of the meatal plug in human and mouse embryos. The meatal plug, or plate, was evident at CS18 as a small group of epithelial cells at the tip of the primary ear canal (Fig. 1A). This plug of cells extended inwards towards the developing middle ear and the extending 1st pharyngeal pouch at CS22 (Fig. 1B). An extended meatus, running parallel to the pharyngeal pouch endoderm and reaching the developing tympanic ring, was evident by CS23 (Fig. 1C). In mice, the meatal plug was apparent as a small group of cells at the edge of the primary canal, which had started to extend alongside the pharyngeal pouch by E13.5 and E14.5 (Fig. 1D,E). Extension in the mouse is angled more anteriorly compared with the human so that the full extension cannot be seen in a single frontal section. The epithelium of the primary canal, endodermal pouch, meatus and skin expressed keratin 5, a marker of undifferentiated epithelium, although expression in the meatus was slightly weaker (Fig. 1F).

To understand the process of extension in more detail we investigated proliferation in the developing human ear canal using Ki67, which labels cells in the active phases of the cell cycle (Scholzen and Gerdes, 2000). Two distinct patterns of expression were observed in the two different parts of the canal at CS23. In the primary canal, proliferation was almost completely restricted to the basal epithelial cells with limited proliferation in the stratified epithelium of the canal walls (Fig. 1G). In contrast, the solid meatal plate had proliferating cells throughout the plate epithelium (Fig. 1H), suggesting a different mode of growth in the two parts of the canal.

In the adult, the more superficial section of the ear canal is supported by elastic cartilage while the deep section is supported by bone. The primary canal has been suggested to form the cartilaginous region of the canal based on histological studies (Michaels and Soucek, 1989). To investigate the position of the junction between the two parts of the canal (primary canal and meatal plug) we compared the different parts of the epithelial canal, as labelled with E-cadherin, with the location of the forming cartilaginous support for the canal, using the early cartilage marker Sox9 (Fig. 1I). The mesenchyme expressing Sox9 was found to lie under the primary canal. In contrast, the area under the meatus part of the canal was not associated with Sox9 expression (Fig. 1I). This suggests that the junction between the two parts of the ear canal may lie at the end of the elastic cartilage region in adult ears.

### The primary canal closes during canal development to form a continuous solid epithelial structure

**Fig. 2.**
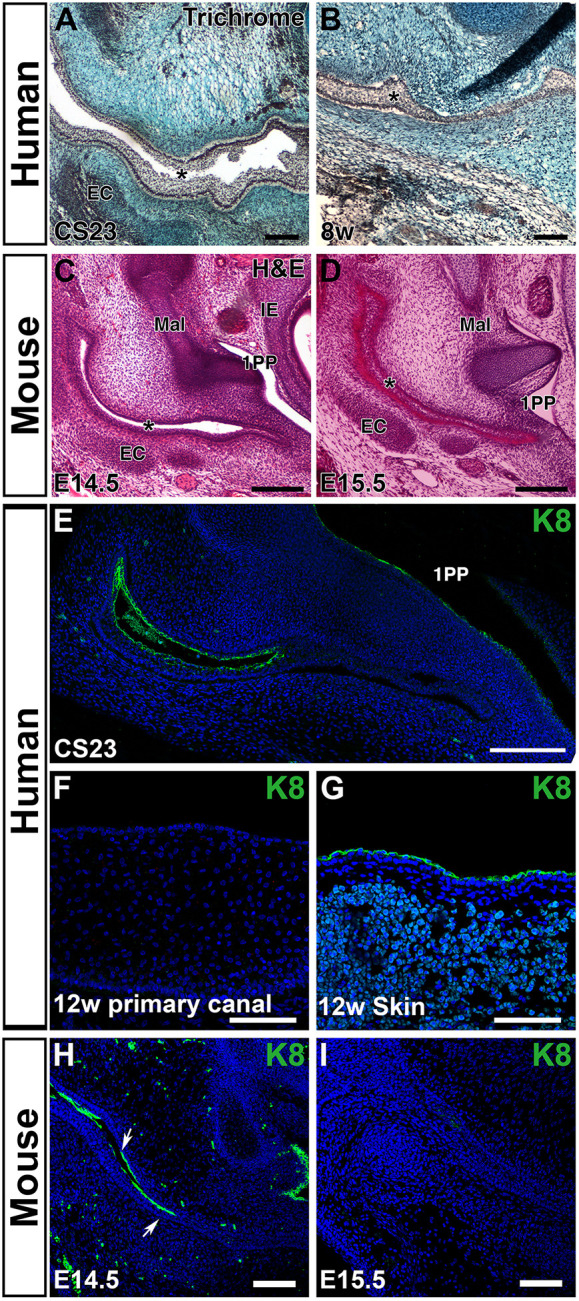
**The primary canal closes during early fetal development.** (A,B) Trichrome staining of the primary canal. Human frontal sections. (A) The primary canal (asterisk) is open at CS23. (B) By late 8 weeks, the canal (asterisk) has turned into a solid epithelial structure. (C,D) A similar closure and fusion is observed in mouse embryos. Eosin/Haematoxylin staining in frontal sections. (C) At E14.5 the canal is open. (D) At E15.5 the canal is closed. (E) Frontal section through the human canal at CS23. Keratin 8 (K8) marks the periderm layer that lines the primary canal, while the meatus does not express K8. (F) Closed primary canal at 12 weeks showing loss of K8-positive cells. (G) The skin still has a K8-positive periderm layer at 12 weeks. (H,I) Mouse frontal sections. (H) At E13.5 the primary canal is lined by K8-positive periderm (arrows). (I) By E15.5 the canal has closed and K8 expression is lost in the canal. Dispersed green cells in the mesenchyme represent autofluorescence from blood cells. EC, ear canal cartilage; Mal, malleus; 1PP, first pharyngeal pouch; IE, inner ear. Asterisks in A-D indicate the primary canal. Scale bars 100 µm in A,B,F-I; 200 µm in C-E.

As the meatal plug reached the tympanic ring, the primary canal was observed to undergo a dramatic change in morphology, with the walls of the canal meeting and fusing leading to loss of the lumen (Fig. 2A-D). This closure and fusion was observed in humans (Fig. 2A,B), mouse (Fig. 2C,D) and guinea pig embryos (Fig. S1), suggesting that loss of the lumen of the primary canal is a conserved feature of mammals. In humans, the closure was evident between CS23 and late 8 weeks of development (Fig. 2A,B). In mice, the closure occurred between E14.5 and E15.5 (Fig. 2C,D), while in guinea pigs it occurred between E28 and E32 (Fig. S1).

During early skin development, the epithelium is covered by a layer known as the periderm that acts as a non-stick coating to prevent adjacent epithelia from sticking to each other. Later on, the cornified layer of skin performs this role and the periderm is no longer needed (Richardson et al., 2014). In mutants with defective periderm, the epithelium can start to adhere, e.g. in the mouth the palatal shelves can become stuck to the tongue, and the upper and lower jaws can fuse together (Richardson et al., 2014). We therefore looked to see whether selective loss of periderm in the primary canal might drive fusion in this area. For this, we selected keratin 8 (K8), as it has been used as an early marker of periderm (Dale et al., 1985; Liu et al., 2013). At CS23, periderm lines the primary canal (Fig. 2E). As might be expected, no K8-positive periderm was associated with the meatal plug. Interestingly, however, once the canal had closed, periderm was no longer evident in the primary canal (Fig. 2F). Loss of periderm was specific to the primary canal as the skin around the canal continued to express K8 at this stage (Fig. 2G). The same loss of K8 was also observed in the mouse between E14.5 and E15.5 (Fig. 2H,I).

### Loss of periderm through apoptosis underlies the closure of the canal

**Fig. 3.**
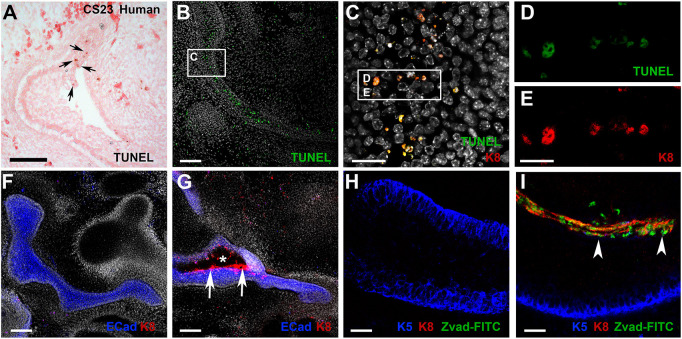
**Periderm is selectively removed by apoptosis during canal closure.** (A) At CS23, as the primary canal starts to close, TUNEL-positive cells (brown) are observed in the human canal epithelium (arrows). (B) As the canal closes in the mouse, TUNEL-positive cells (green) are observed at high levels in the centre of the closed canal. (C) High-power magnification image of the murine closed canal (see rectangle in B). (D,E) The TUNEL-positive cells (green) overlap with the periderm, as shown by co-expression with keratin 8 (K8) (red) (see rectangle in C). (F-I) Culture of the murine ear canal in explant slices. E13.5 cultured for 3 days. (F,H) In control cultures, the primary canal had closed and fused during this time frame. (F,G) E-cadherin (blue), K8 (red) and DAPI (white). (G) In the presence of the inhibitor, the ear canal fails to fuse successfully, with K8-positive cells (arrows) still present in the open canal (asterisk). (H,I) Keratin 5 (blue) labels the basal epithelium to outline the canal. FITC-Z-Vad-FMK (green) labels cells that have been inhibited from undergoing apoptosis. (H) In the controls, K8-positive cells (red) are no longer present and there are no cells with active caspase expression (green). (I) In the inhibitor-treated cultures, K8-positive cells (red) remains at high levels at the centre of the canal (arrowheads), overlapping with the cells that have been prevented from undergoing apoptosis (green). Scale bars: 100 µm in A,B; 50 µm in C; 70 µm in F,G; 25 µm in D,E,H,I.

In the human and mouse, closure of the canal coincided with the presence of a large number of apoptotic cells within the primary canal, as revealed by TUNEL staining (Fig. 3A,B). The TUNEL-positive cells overlapped with K8 expression in the mouse, highlighting that it was specifically the periderm that was undergoing cell death (Fig. 3C-E). Loss of the periderm by apoptosis may therefore provide a mechanism to allow fusion of the sides of the primary canal. To test this hypothesis, we established a novel technique to culture the ear canal. Explant slices containing the murine ear canal were cultured for 3 days in the presence of Z-VAD-FMK, a pan-caspase inhibitor that has previously been shown to block apoptosis in culture (Teshima et al., 2016). Ear canal slices were cultured from E13.5, prior to closure of the canal. After 3 days, the control cultures showed a closed primary canal, with confluent expression of the epithelial marker E-cadherin, mimicking development *in vivo* (Fig. 3F) (*n*=10/10 complete closure). No evidence of periderm, as indicated by K8 expression, was observed (Fig. 3F). In contrast, in the cultures with the apoptosis inhibitor the canal remained open in patches, which were lined by K8-positive cells (Fig. 3G) (*n*=10/10 incomplete closure). The extent to which the canal remained open varied, even within a culture, suggesting incomplete inhibition of cell death, but this provided an interesting internal control, with the open regions of canal always lined with K8 cells (Fig. S2). In order to label cells that had been prevented from undergoing apoptosis due to the presence of the inhibitor, the marker FITC Z-VAD-FMK was added at the end of the culture period. As expected, no labelling was observed in the control canal epithelium, as apoptosis has finished by this timepoint (Fig. 3H). However, in the inhibitor-treated cultures, the K8-positive cells in the centre of the canal showed a high level of overlap with FITC, confirming that it is the periderm that would normally undergo apoptosis (Fig. 3I).

### Premature closure of the canal in mice with periderm defects

**Fig. 4.**
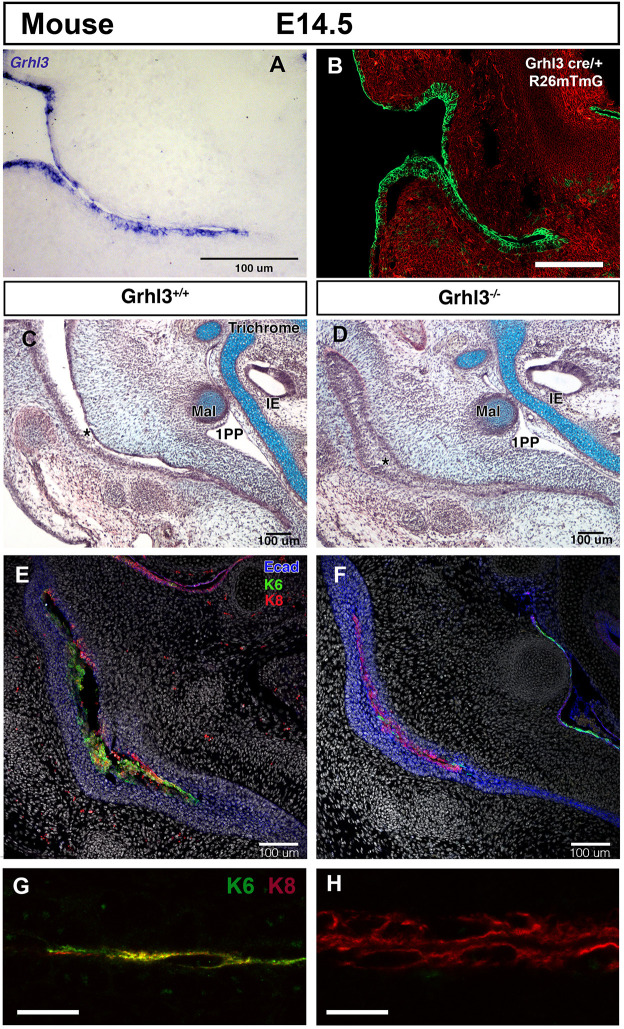
**Defects in the periderm layer lead to precocious closure of the primary canal.** (A-H) Mouse E14.5 frontal sections. (A) *In situ* hybridisation of Grhl3 in the epithelium of the developing mouse ear canal at E14.5. (B) Grhl3cre mTmG, showing expression of Grhl3 in green throughout the ear canal epithelium. (C,D) Trichrome staining of the ear canal. (E-H) IF for keratin 6 (green), keratin 8 (red), E-cadherin (blue) and DAPI (white). (C,E,G) Wild-type mouse. (D,F,H) Grhl3 null littermate. (C) The primary canal is open (asterisk) and (E) lined by cells expressing the periderm markers K6 and K8 (green and red, respectively). (D) In the mutant, the primary canal is prematurely closed (asterisk) and (F) the periderm marker K6 is absent. K8-positive cells (red) are still present at the centre of the closed canal, highlighted by E-cadherin (blue). (G) Close up of the wild-type canal. The periderm is double stained with K6 and K8. (H) Close up of the Grhl3 null canal. Only K8-positive cells are present. IE, inner ear; Mal, malleus; 1PO, first pharyngeal pouch. Scale bars: 100 µm in A-F; 12.5 µm in G,H.

Having shown that retention of periderm prevented canal closure, we investigated whether loss of periderm would disrupt normal canal development. To do this, we analysed mice carrying a loss of function allele of Grhl3*,* encoding the grainyheadlike 3 transcription factor. Grhl3 is required for the development and repair of the epithelial barrier layer (Yu et al., 2006). Mutations in human GRHL3 are associated with oral clefting leading to Van der Woude syndrome (Peyrard-Janvid et al., 2014) and non-syndromic clefting cases. Grhl3 mouse mutants have abnormal periderm, with very reduced expression of keratin 6 (K6, another periderm marker) and fusions of the oral epithelium (Peyrard-Janvid et al., 2014). The periderm appears to form in the mutants, based on expression of the early periderm marker keratin 17, but the cells are larger and have a spikey cell surface (Kashgari et al., 2020). Grhl3 is expressed early in the posterior neural tube and tail bud region (De Castro et al., 2018), and later throughout the developing skin, including the epithelium of the primary ear canal (Fig. 4A). This pattern is largely recapitulated by lineage tracing using a Grhl3-cre allele with the mTmG reporter (Fig. 4B) (Camerer et al., 2010). In Grhl3-null mice at E14.5, the primary ear canal had already closed (*n*=5), in contrast to wild-type littermates where the canal was still open (Fig. 4C,D) (*n*=3 littermates, *n*=30 non-littermate wild type). At E13.5, the mutant canal had formed but was already showing signs of fusion, 2 days earlier than wild-type mice (Fig. S3, *n*=3). At E14.5, the open wild-type canal expressed both K8 and K6 at high levels, with expression largely overlapping for these two markers (Fig. 4E,G). In contrast, in the closed mutant canal, expression of K6 was absent, but K8 was still robustly expressed in the cells at the centre of the closed canal (Fig. 4F,H). This suggests that a rudimentary, K8-expressing periderm might still be present in the mutants but was not functioning to repel adjacent epithelial cells. Loss of a functioning periderm therefore leads to premature ear canal closure.

### Terminal differentiation triggers opening of the canal

**Fig. 5.**
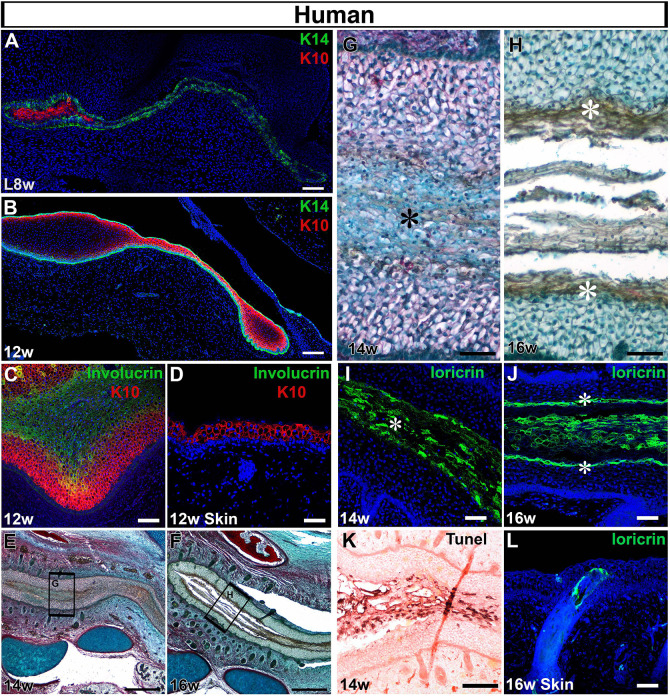
**Opening of the canal involves a precocious programme of differentiation.** (A-L) Frontal sections through human fetal samples. (A-C,E-K) Developing ear canal. (D,L) Adjacent cranial skin. (A) At late 8 weeks, the closed canal comprises basal K14 cells (green) and more differentiated K10 cells (red) at the centre. DAPI nuclear stain (blue). (B) By 12 weeks the whole canal is lined with K14- (green) and K10-positive (red) cells. (C) The central cells of the canal expressed the granular layer marker involucrin (green), adjacent to the keratin 10 layer (red). (D) The skin at the same stage had not yet formed an involucrin-positive layer (green), above the K10-positive cells (red). (E-H) Trichrome-stained sections. (E) The ear canal is starting to open by 14 weeks. (F) A clear gap, filled with sloughed off cells, is evident at 16 weeks. (G) High-power magnification view of boxed are in G, highlighting distinct morphology of the cells at the centre of the canal (asterisk). (H) High-power magnification view of boxed are in F, highlighting distinct morphology of the cells lining the open canal (asterisk), with sloughed off cells in the centre. (I) At 14 weeks, the cells at the centre of the primary canal expressed locricrin (green), indicating terminal differentiation of the canal epithelium. DAPI nuclear stain (blue). Asterisk labels central cells. (J) As the canal opened, its sides were labelled with locricrin (asterisks), which also labelled the sloughed off cornified layer. (K) At 14 weeks the locricrin-expressing layer was associated with high levels of TUNEL staining (brown), indicating programmed cell death (Eosin counterstain). (L) Loricrin is not expressed in the skin by 16 weeks but some expression is associated with forming hair follicles. DAPI nuclear stain (blue). Scale bars: 100 µm in A,C,K; 150 µm in B; 500 µm in E,F; 50 µm in D,G-J,L.

Closure of the primary canal creates a single solid epithelial structure along the whole length of the ear canal that then needs to open to create a functional tube. To understand how opening occurred, we followed the pattern of differentiation leading up to opening in human fetal samples. In the mouse at E14.5, the embryonic epidermis is constructed of a K14-positive basal layer, an intermediate layer and a periderm. By E15.5, the intermediate layer stops proliferation and turns on K10, and is then referred to as the spinous layer (Koster and Roop, 2004). K10 is associated with committed suprabasal cells and withdrawal from the cell cycle. In human embryos, cells of the intermediate layer have been identified at 9-10 weeks, with only basal and periderm cells observed before this point (Dale et al., 1985). At late 8 weeks, we observed expression of K14 in the basal layers of the canal and throughout the meatus, with K10 expressed in between the K14 layers of the closed primary canal (Fig. 5A). This expression of K10 is earlier than has been reported in the skin and suggests premature differentiation in the canal. By 12 weeks the intermediate layer of K10 cells was found throughout the ear canal (Fig. 5B).

The centre of the closed ear canal, and tip of the meatus, were full of K14K/10-negative cells that expressed involucrin, a marker of the granular layer during epidermal differentiation (Fig. 5C). Interestingly, no involucrin was observed in the cranial skin next to the ear canal at this time in the same fetal sample, again suggesting precocious development of the canal in contrast to the neighbouring skin (Fig. 5D). By 14 weeks, the ear canal was closed along the majority of its length (Fig. 5E,G), whereas by 16 weeks the ear canal had opened (Fig. 5F,H). At 14 weeks, the cells at the centre of the canal had a distinctive morphology (Fig. 5G) and expressed the terminal differentiation marker loricrin, which labels the cornified layer (Fig. 5I). Expression of loricrin therefore preceded opening of the canal. By 16 weeks loricrin-expressing cells were found at the edges of the open canal and in the sloughed off cells in the canal centre (Fig. 5H,J). Again, this expression of loricrin was earlier than in the neighbouring cranial skin, where loricrin was expressed only in developing hair follicles at 16 weeks (Fig. 5L). Interestingly, the terminal differentiated cells expressing loricrin stained positive for TUNEL, indicating high levels of programmed cell death (Fig. 5K).

### Development can be used to suggest mechanisms underlying ear canal defects in patients

**Fig. 6.**
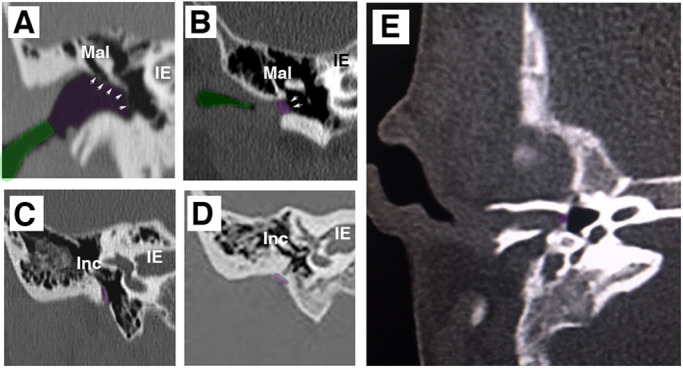
**Scans of canal aplasia and atresia in patients.** CT scans of patients. (A) Normal ear. (B-E) Patients with ear canal defects. (A) Coronal section through the ear showing a normal ear with a patent bony (purple) and cartilaginous (green) canal. The tympanic membrane is visible and outlined with arrows. (B) There is a stenotic lateral ear canal (green) and a medial meatal plug (purple). Arrows indicate the medial aspect of the metal plug at the tympanic annulus. (C,D) Patients have no evidence of a canal and the middle ear is closed off by a bony atretic plate (purple), which can be thin (C) or thick (D). (E) Patient with a closed ear canal. The bone still forms around the canal, suggesting that epithelial tissue is present. The primary canal is open near the pinna. IE, inner ear; Inc, incus; Mal, malleus.

Canal defects are variable, affecting the bony and cartilaginous canals to different extents in different patients. The air-filled canal is evident as darkened areas on a CT scan, allowing the canal to be outlined (Fig. 6A, normal ear). Ear canal defects can be divided into categories depending on severity (Schuknecht, 1975). Type I involves narrowing of the ear canal but sound can still pass through the canal to the middle ear (Fig. 6B). In such cases, it can be presumed that the auditory meatus was able to extend normally but a problem occurred during opening, leading to a narrow canal. This phenotype is therefore associated with later defects in ear canal formation. Type II defects can be divided into two groups: patients with no canal and patients with some evidence of a canal that ends in a shallow blind pouch and an osseous ‘atretic plate’. Examples of Type II defects are shown in Fig. 6C-E. In Fig. 6C,D, no canal is evident and the tympanic membrane is replaced by a bony atretic plate, which can be thin (Fig. 6C) or thick (Fig. 6D). Here, it would be predicted that the epithelial meatal plate failed to extend towards the middle ear and that, in the absence of a canal, bone has been deposited where the ear drum should have formed. In the patient shown in Fig. 6E, the primary canal is evident but truncated. Interestingly, in this case the bone has still formed the walls around a canal. This suggests that the epithelial canal did reach the middle ear, shaping the hard tissue that was laid around it, but failed to open. Epithelial tissue reaching the middle ear would therefore be predicted to be present in patient E but not in patients C and D. Understanding how the human ear canal develops, therefore, can explain some of the underlying mechanisms at work in cases of atresia, and can highlight whether or not canal reconstruction is recommended.

## DISCUSSION

**Fig. 7.**
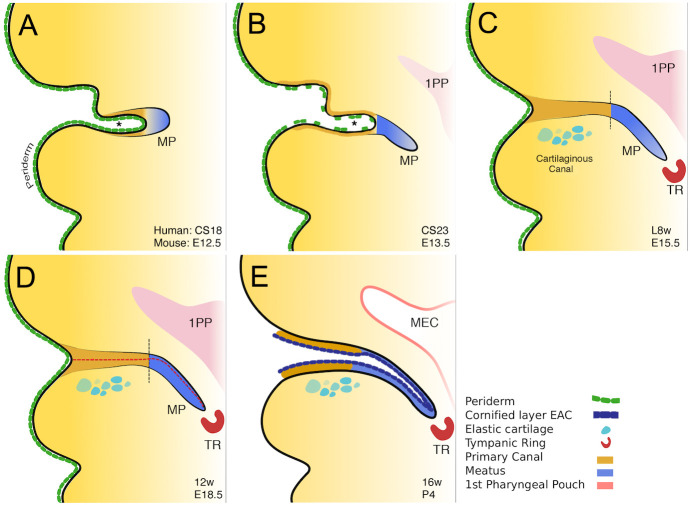
**Schematic of mammalian ear canal development.** (A) Invagination of the primary canal (asterisk) with a meatal plug (MP) at its tip. (B) Extension of the meatus towards the forming middle ear and selective loss of periderm in the primary canal. (C) Closure of the primary canal and further extension of the meatal plug to reach the tympanic ring (TR). (D) Loricrin expression (red dashed line) marks the site of opening of the canal. Vertical black dashed lines between brown and blue regions in C and D represent the two distinct parts of the ear canal. (E) The whole external ear canal opens, with the upper/rostral wall of the meatal plate forming the outer surface of the ear drum, and the open canal lined by a cornified layer. 1PP, first pharyngeal pouch; MEC, middle ear cavity. Stages in schematics represent approximate age in human embryonic and fetal stages (top line), and in days of embryonic development (E) and postnatal development (P) in the mouse (bottom line).

In this article, we show that the mammalian ear canal is made up of two parts with a very different mode of development. The more superficial part is originally formed as an open invagination (primary canal) that closes and later reopens. In contrast, the deeper part is formed by a solid meatal plug that extends as a thin sheet of epithelial cells which then opens up (Fig. 7). The closure of the primary canal at early stages of development to form a single solid structure was previously noted from histology sections (Nishimura and Kumoi, 1992). The early closure probably explains why this two-mode system has not been well explained in previous literature, where the whole canal is often referred to as a meatus.

Proliferation in the meatal plug was fairly uniform throughout the structure but it is possible that orientated cell division may play a role in extension of this tissue towards the middle ear. In contrast, proliferation in the primary canal was restricted to the basal layers, in a similar manner to the skin (Fuchs, 2007). This would lead to stratification and apposition of the tissues on either side of the canal. Early fusion of the sides of the canal is prevented by the presence of a superficial layer of periderm, as indicated by expression of K8 and K6. In the Grhl3-null mutants, where the periderm is defective, the primary canal forms but fuses prematurely. During and immediately after fusion, high levels of apoptosis were evident in both human and mouse embryos, agreeing with previous reports that showed apoptosis in the mouse ear canal at E15.5 (Nishizaki et al., 1998). Cell death in this case appears to specifically remove the periderm layer from the canal and allow the canal epithelium to come together. Inhibition of cell death in culture, led to maintenance of K8-expressing periderm cells, preventing canal closure and fusion. In the Grhl3-null mice, expression of K6 was lost. Loss of K6 has been shown to alter the migratory capacity of keratinocytes, by regulating cell matrix and cell-cell adhesion (Wang et al., 2018). The migratory capacity of epithelial cells might therefore be enhanced in the absence of Grhl3, further aiding closure of the primary canal.

Fusion *in vivo* is seamless, with no evidence of a join. It is unclear why closure and later reopening is required for formation of this structure. One possibility is that closure protects the forming middle ear. A similar closure, fusion and reopening is observed during eyelid development. Here, the two eyelids (upper and lower) cover the developing eye, fuse together, and then detach from each other at the end of eye development to allow the eyes to open (Heller et al., 2014).

This two-part development of the canal is not restricted to humans but was observed in mice and guinea pig embryos, and therefore appears a general feature of mammals. Canal development is very different from that shown recently in chick embryos where the canal develops from the primary canal, which remains open at all stages (Furutera et al., 2017). No meatal plug/plate has been observed in chick or reptile embryos, suggesting that this is a novel mammalian structure, agreeing with the view that the mammalian and non-mammalian tympanic membrane are not homologous (Furutera et al., 2017; Kitazawa et al., 2015; Tucker, 2017). Certainly, the way the outer layer of the tympanic membrane develops in mammals and non-mammalian tetrapods is very different.

Our results show that the position of the opening of the canal could be predicted by the expression of loricrin, which marked the terminally differentiated cells (Fig. 5). Opening therefore appeared as a result of a wave of differentiation with cell death again playing a role in canal development, this time to remove terminally differentiated and sloughed off epithelial cells to create an opening. Previously, cell death was not found to be associated with opening in the mouse, but it was suggested that cell death might be associated with terminal differentiation of the ear canal epithelium (Nishizaki et al., 1998). Interestingly, the canal developed precociously when compared with the neighbouring skin. This is unexpected as the canal is derived from an invagination of the surface ectoderm and so might be predicted to follow a similar developmental time scale for differentiation. The signals that induce early differentiation are unknown and would be an interesting future study. The canal opened between 14 and 16 weeks, agreeing with the work of Nishimura and Kumoi (1992) and Anson et al. (1955) , but disagreeing with Michaels and Soucek (1989), who suggested opening occurred at 18 weeks, at the same time as cornification of the skin.

Overall, we show that ear canal development is far from simple, with complex epithelial dynamics that would not be predicted from the final shape. Defects can occur at a number of stages. For example, the auditory meatus might fail to extend and never reach the middle ear, or the canal might reach the middle ear but not open, or the canal might open but not expand to create a fully patent tube. This new knowledge allows a reassessment of ear defects in patients and provides key information for ear canal reconstruction.

## MATERIALS AND METHODS

### Human embryo samples

Human embryonic and fetal samples were sourced from the Human Developmental Biology Resource tissue bank. Carnegie stages (CS) 17, 18, 20, 22 and 23 (corresponding to 42, 44, 49, 53 and 56 post conception days, respectively) and late 8, 12, 14, 16, 17 and 18 gestational week fetal samples were used in this study (see Table 1). *n*=1-3 for each stage.

**Table 1.**
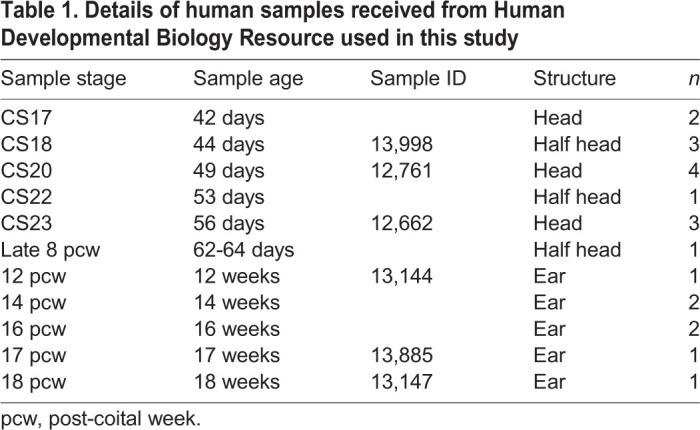
**Details of human samples received from Human Developmental Biology Resource used in this study**

### Mouse and guinea pig samples

Mouse embryos were collected from time mated CD1 crosses. Day 0.5 was considered midday on the day that a plug was found. Pregnant *Cavia porcellus* females were obtained from Harlan. Females were housed with a male at the time of giving birth. The following day was assumed to be embryonic day 0. Pregnant females were sacrificed at 28 and 32 days of embryonic development. Each female had three pups. Grhl3^cre/+^ mice were mated to R26mTmG reporter mice to generate foetuses that provide a readout of Grhl3 lineage (Camerer et al., 2010). Grhl3-null foetuses (*Grhl3*^−/−^) were generated by intercross of *Grhl3*^+/−^ heterozygotes and genotyped by PCR of genomic DNA (Yu et al., 2006; De Castro et al., 2018).

### Trichrome staining

Embryos were fixed in 4% paraformaldehyde and dehydrated through an ethanol series before embedding in paraffin wax. Human fetal samples were decalcified before processing in EDTA. Sections were cut on a microtome at 12 μm (human) or 8 μm (mouse and guinea pig) and slides were stained with either a trichrome stain (Haematoxylin, Alcian Blue and Sirrus Red) or with Haematoxylin and Eosin. Sections were photographed using a Nikon eclipse 80i microscope.

### Immunohistochemistry/immunofluorescence

Wax sections were dewaxed, rehydrated, treated with DAKO antigen retrieval solution at 95°C for 30 min followed by 20 min at room temperature. The slides were then incubated with rabbit anti-keratin 14 (1:200; Covance, 905501), mouse anti-E-cadherin (1:250; Abcam, ab76055), rabbit anti-Sox9 (1:400; Millipore, ab5535), mouse anti-keratin 10 (1:300; Abcam, ab76318), rabbit anti-keratin 5 (1:300; Covance, PRB-160P), rat anti-keratin 8 (1:100; TROMA-1), mouse anti-involucrin (1:200; SY7), rabbit anti-loricrin (1:200; Biolegend, PRB-145P) and rabbit anti-Ki67 (1:200: Abcam, ab16667) overnight at 4°C. Primary antibodies to keratin 8 and involucrin were kindly provided by the lab of Fiona Watt (King's College London, UK). The sections were then washed and incubated in goat anti-rabbit biotin (1:500; DAKO) or goat anti-mouse biotin (1:500; DAKO). Amplification was performed using Streptavidine_HRP (PerkinElmer) then developed using DAB with Nickel (Vector SK4100), counterstained with Eosin. For immunofluorescence, sections were incubated in Alexafluor donkey anti-mouse 488 (1:500; Invitrogen, A11001), Alexafluor donkey anti-rabbit 568 (1:500; Invitrogen, A10042) and Alexafluor donkey anti-rat 647 (1:500; Invitrogen, A21247) for 2 h at room temperature. Sections were mounted with fluoroshield with DAPI (Sigma-Aldrich, SLBV4269) and imaged with a Leica TCS SP5 confocal microscope. To test each antibody, controls were performed where the primary antibodies had been omitted in order to confirm specific staining.

### TUNEL

A TUNEL assay was performed following the manufacturer's instructions (Millipore, S7100). Staining was carried out using DAB (Vector SK4100) or TSA-Cy3. As a positive control, the inner ear was studied, as clear apoptosis has previously been shown in this neighbouring structure (Nishikori et al., 1999).

#### Explant culture

Animal tissue was collected from wild-type mice of CD1 strain housed in the Biological Services Unit in New Hunts House at King's College London. Embryonic heads at E13.5 were dissected out and chopped on a McIlwain Tissue chopper at 250 μm (Alfaqeeh and Tucker, 2013). These live slices were then sorted and slices with the primary ear canal were selected for culture. Explants were placed on permeable membranes (BD Falcon cell culture inserts, pore size 0.4 μm) and floated over culture medium consisting of Advanced Dulbecco's Modified Eagle Medium F12 (DMEM F12) (Invitrogen) supplemented with 1% Glutamax (Invitrogen) and 1% penicillin-streptomycin, and were cultured in a 37°C and 5% CO_2_ incubator for 3 days.

Pan-caspase inhibitor Z-VAD-fmk (Promega, G7231) was used for the *in vitro* inhibition of apoptosis at a concentration of 250 µM, based on previous dose-dependent experiments performed during culture of other craniofacial tissue (Teshima et al., 2016). The experimental groups were compared with ear canal slices cultured in DMSO at the same concentration (1.25%). *In situ* CaspACE FITC-Z-VAD-FMK marker (Promega, G7461) was added to the culture medium in the last day of culture for 30 min under the same normal culture conditions. Cultured canals were then fixed for 2 h at room temperature and washed in PBS. Permeabilisation was performed with PBS Triton 0.5% for 1 h followed by trypsinisation for 5 min on ice. Blocking unspecific proteins was carried out with blocking solution for 2 h. Primary antibodies were incubated overnight at 4°C. Secondary antibodies were incubated at a concentration of 1:500 (Alexa Fluorophore, Invitrogen) overnight. All cultures were then analysed by confocal laser-scanning microscopy (Leica TCS SP5).

#### *In situ* hybridisation for Grhl3

*In situ* hybridisation was performed as described by Fons Romero et al. (2017). A pGEM-T easy plasmid (A1360, Promega) was linearised with SalI (R6051, Promega) overnight at 37°C and purified with Monarch PCR&DNA cleanup kit (T1030G, NEB). 1 µg of the linear DNA was used for an *in vitro* transcription with T7 RNA polymerase (P2075, Promega) to synthesise the Grhl3 probe.

#### Study approval

Anonymised patient scans were provided by S.C. The project ‘An analysis of middle and external ear defects in patients attending the specialist microtia and atresia clinic’ was registered with Guy's and St Thomas' Trust R&D (RJ115/N103). Scans formed part of the normal patient assessment at the clinic. Ethical approval for the use of human embryonic and fetal samples were provided by the Human Developmental Biology Resource (Project 200362, An analysis of external ear development).

For mouse and guinea pigs, adults and embryos were culled using a schedule 1 method as approved by the UK Home Office. Home Office Establishment, Project and Personal licences were all in place for the breeding of transgenic lines used in this research.

## Supplementary Material

Supplementary information
