# Supplementary material for: Epithelial dynamics shed light on the mechanisms underlying ear canal defects
Source: Development. 2020 Dec 14;147(23):dev194654. doi: 10.1242/dev.194654 (PMC7758633; doi:10.1242/dev.194654)
Supplement: Supplementary information [file develop-147-194654-s1.pdf]

Figure S1:
Collapse of the primary canal during guineapig ear development.
(A) Embryonic day (E) 28, the canal is open.
(B) Embryonic day (E) 32, the canal is closed. White arrows highlight the extending meatal plug. Asterix marks the primary canal.
Scale bar in A,B = 250µm.

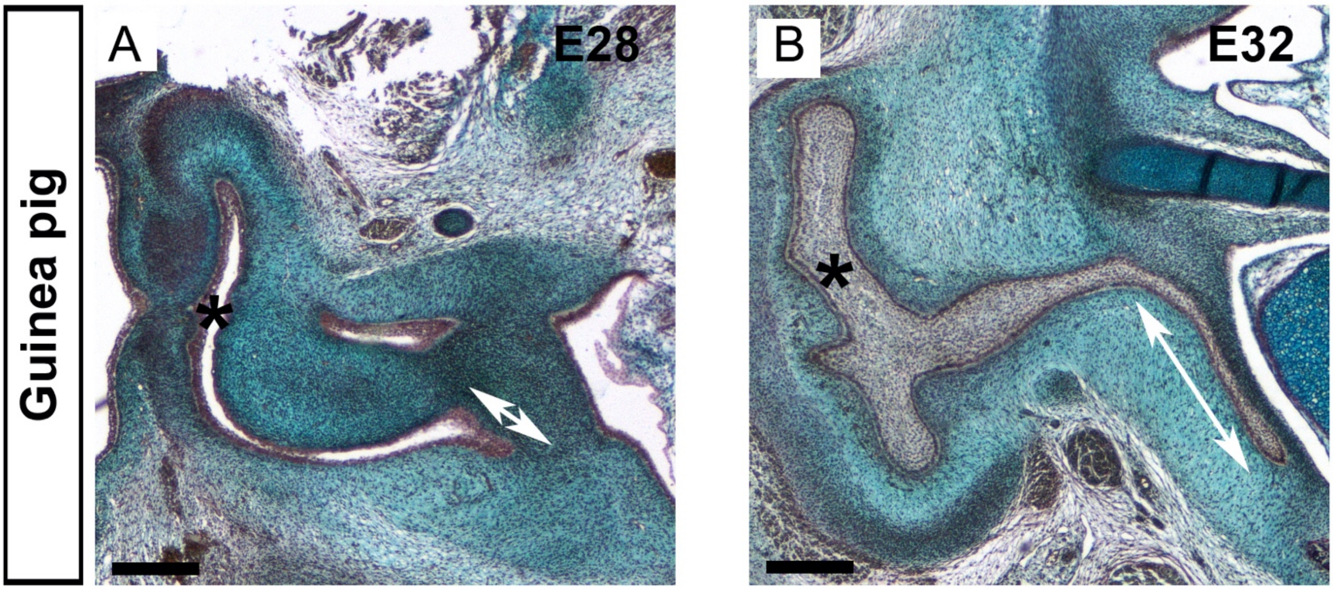

Figure S2:
Prevention of canal closure by inhibition of apoptosis.
(A) Cultured ear canal from E13.5 for three days. Closed canal (asterix) expressing Ecadherin (green), Keratin 10 (blue) with no Keratin 8 (red). (B) After culture with ZVAD the canal is closed in places (asterix) as in controls, but remains partially open in neighbouring regions, associated with the retention of Keratin 8 (red) expressing cells (arrowhead).
Scale bar in A,B = 30µm.

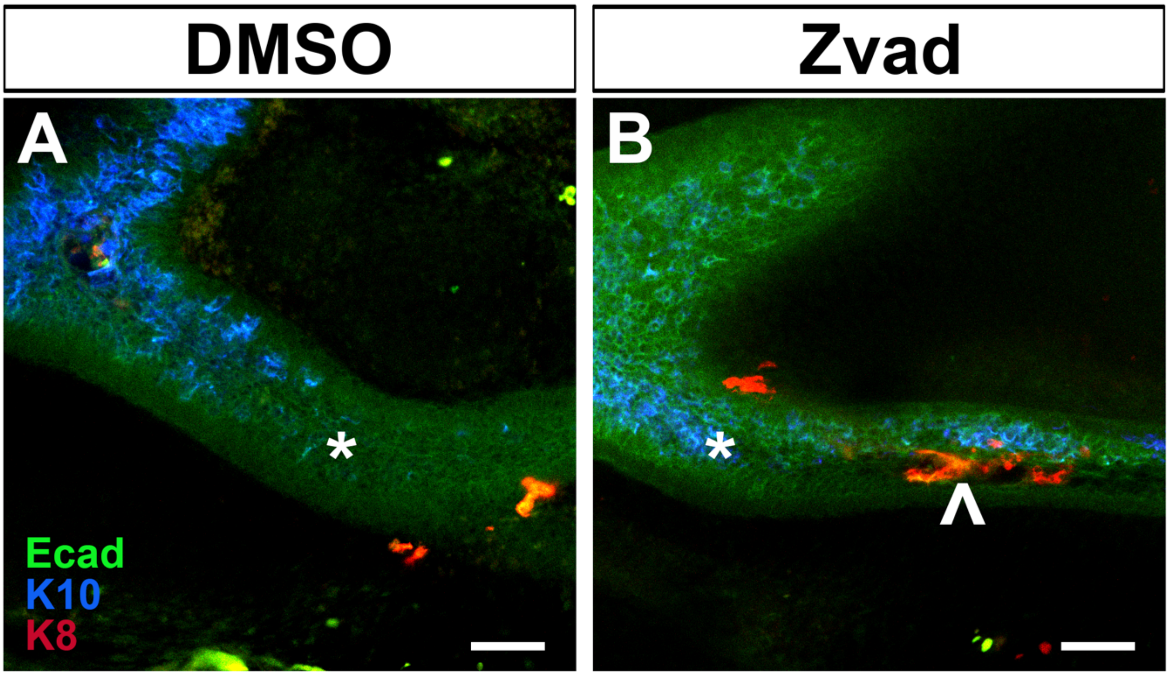

Figure S3:
The primary canal forms but closes prematurely in Grhl3 mutants at E13.5.
(A,B) Control littermate showing extending open primary canal ending in a meatal plug.
(C,D) Grhl3 mutant showing formation of the primary canal but fusion near the meatal plug (arrow in D).
Scale bars (A-D): 100µm.

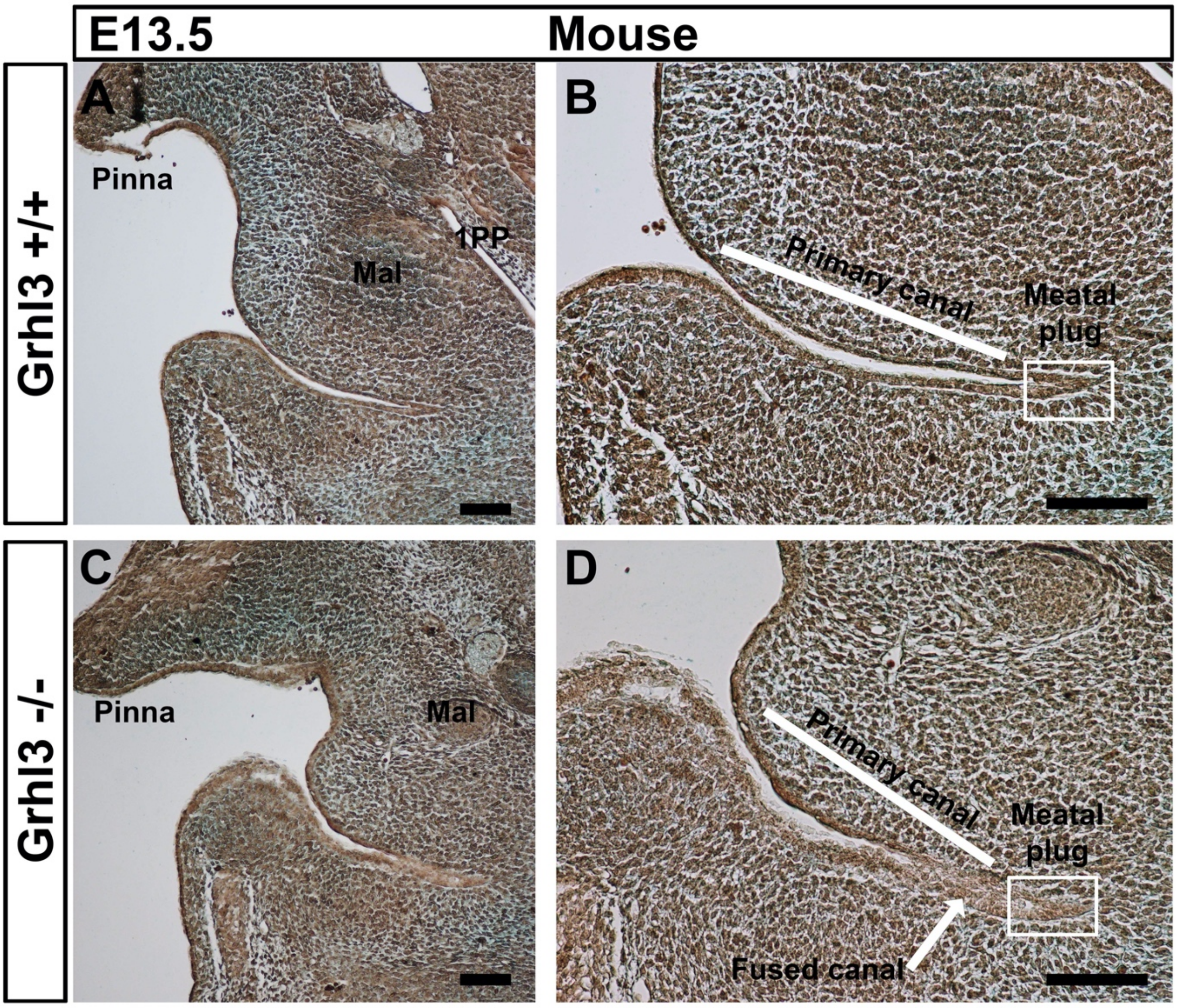

Development • Supplementary information
